# Prevalence of depression, anxiety, fatigue, and headache before and after long COVID onset: a case–control study in the total population of Region Stockholm

**DOI:** 10.1186/s12916-025-04498-w

**Published:** 2025-11-14

**Authors:** Sebastian Lindblom, Pia Lindberg, Gunnar Ljunggren, Seika Lee, Marta A. Kisiel, Iryna Kolosenko, Predrag Petrovic, Myrto Sklivanioti Greenfield, Caroline Wachtler, Artur Fedorowski, Åsa M. Wheelock, Axel C. Carlsson

**Affiliations:** 1 Division of Family Medicine and Primary Care, Department of Neurobiology, Care Sciences and Society, Karolinska Institutet, Huddinge, Sweden; 2 Women’s Health and Allied Health Professionals Theme, Karolinska University Hospital, Stockholm, Sweden; 3 Division of Physiotherapy, School of Health, Care and Social Welfare, Mälardalen University, Västerås, Sweden; 4 Division of Immunology and Respiratory Medicine, Department of Medicine Solna, Karolinska Institutet, Stockholm, Sweden; 5 Department of Respiratory Medicine and Allergy, Center for Molecular Medicine, Karolinska University Hospital, Solna, Sweden; 6 Academic Primary Health Care Centre, Region Stockholm, Stockholm, Sweden; 7 Department of Medical Sciences, Occupational and Environmental Medicine, Uppsala University, Uppsala, Sweden; 8 Department of Clinical Neuroscience, Centre for Psychiatry Research, Karolinska Institutet, Stockholm, Sweden; 9 Department of Clinical Neuroscience, Center for Psychiatry Research and Center for Cognitive and Computational Neuropsychiatry, Karolinska Institutet, Stockholm, Sweden; 10 Consultation Liaison Unit, Psychiatry Southwest Stockholm, Stockholm, Sweden; 11 Department of Cardiology, Karolinska University Hospital, Stockholm, Sweden; 12 Department of Medicine, Karolinska Institutet, Stockholm, Sweden

**Keywords:** Post-Acute Sequelae of SARS-CoV-2 infection (PASC), Post-Acute COVID-19 Syndrome (PACS), Non-hospitalized, Mental health, Primary health care, ICD-10 diagnoses

## Abstract

**Background:**

Post-acute sequelae of SARS-CoV-2 infection, or long COVID, include diverse symptoms and remain a major concern worldwide. This study investigates the occurrence of depression, anxiety, fatigue, and headache 1 year prior to the COVID-19 pandemic (2019), 12 months prior to, and 6 months after long COVID diagnosis in individuals diagnosed with long COVID and matched population-based controls.

**Methods:**

This case–control study included nonhospitalized individuals diagnosed with long COVID compared with controls without long COVID, matched by age, sex, and neighborhood socioeconomic status. Data were collected from the Stockholm Regional Health Care Data Warehouse (VAL), including diagnoses in 2019, 12 months before, and 6 months after the long COVID diagnosis. Conditional logistic regression was used to calculate odds (OR) ratios and 99% confidence intervals (CI).

**Results:**

A total of 5589 cases (mean age: 47 years, 69% female) and 47,561 controls were included. Individuals with long COVID had a higher pre-pandemic frequency of the following diagnoses: depression (women: *OR* 1.57 (1.26–1.97), men: *OR* 1.40 (0.88–2.23)), anxiety (women: *OR* 1.65 (1.41–1.93), men: *OR* 2.10 (1.56–2.84)), fatigue syndrome after viral infection (women: *OR* 1.96 (0.86–4.48), men: *OR* 2.22 (0.29–17)), and headache (women: *OR* 2.45 (1.96–3.05), men: *OR* 2.89 (1.86–4.50)). Individuals with long COVID also had a higher frequency of these diagnoses 12 months before and 6 months after the long COVID diagnosis was made, regardless of sex.

**Conclusions:**

Individuals with long COVID had a higher prevalence of depression, anxiety, fatigue, and headache both before and after being diagnosed with long COVID compared with controls without long COVID. The findings suggest an association between mental health vulnerabilities and long COVID, while the frequency of registered mental health diagnoses remained largely similar after the long COVID diagnosis.

## Background

The COVID-19 pandemic, caused by the severe acute respiratory syndrome coronavirus 2 (SARS-CoV-2), has resulted in a global health crisis with profound and long-lasting effects on individuals and healthcare systems [1]. While most patients recover from the acute COVID-19 infection, a significant number of individuals experience symptoms that persist for months or years, described as post-acute sequelae of SARS-CoV-2 infection (PASC) or long COVID [2]. Long COVID is defined as having persistent symptoms > 12 weeks after the acute infection, which cannot be explained by an alternative diagnosis [3–6]. The range of symptoms often experienced by patients includes fatigue, post-exertional malaise, brain fog, cognitive dysfunction, anxiety and depressive symptoms, dizziness, sensorimotor symptoms, gastrointestinal symptoms, palpitations, dyspnea, chronic cough, and chest pain [7, 8], ultimately impacting the overall health-related quality of life [9, 10].

It is estimated that around 10–30% of people previously infected by SARS-CoV-2 are affected by long COVID [2, 11], with the frequency appearing to have decreased in more recent times. However, longitudinal studies have reported numbers as high as 46% of people diagnosed with COVID-19 still having persistent symptoms after 12 months [12, 13]. The variation in numbers is linked to the uncertainties and complexity surrounding the precise diagnostics of long COVID. While long COVID today is recognized as a single condition with multiple distinct phenotypes, its pathophysiology remains unclear [14]. Evidence points to a multifactorial explanation as multiple organs are involved [15], and a combination of direct viral-specific pathophysiological damage, immune dysregulation, microvascular injury, and inflammation in response to acute infection, which may persist and lead to chronic inflammation, exacerbating long-term symptoms [16]. Further, persistent viral reservoirs, aberrant immune responses, and post-viral autonomic dysfunction have all been hypothesized as contributing factors [17]. Importantly, persistent symptoms are not unique to COVID-19 but have also been observed across a wide range of medical conditions, suggesting the involvement of potential transdiagnostic mechanisms [18]. Finally, researchers have argued that different mechanisms may underlie persistent symptoms in individuals with varying infection severity, even though current long COVID criteria do not distinguish between mild and severe cases. Conversely, given that functional neurological disorder often begins after a noxious sensory event, it is expected that some individuals develop functional symptoms following COVID-19 infection. The association between long COVID and functional disorders remains a topic of ongoing debate [19–21].

Although long COVID is recognized as a complex multisystem disorder, the interaction between preexisting mental health conditions and long COVID remains underexplored. Mental health-related diagnoses and associated symptoms such as depression, anxiety, fatigue, and headache are commonly reported symptoms in long COVID literature, have a substantial impact on quality of life, and are highly relevant to clinical management [7, 22]. While recent evidence indicates that preexisting mental health conditions constitute a risk factor for long COVID [23, 24], it remains unclear how these diagnoses and symptoms evolve over time in relation to long COVID. Understanding the relationship between mental health-related diagnoses and associated symptoms, and long COVID, is essential, as mental health conditions could either be a predisposing factor, an exacerbating component, or a consequence of long COVID.

As attempts to characterize the clinical profile of long COVID progress, few studies have examined the prevalence and trajectory of mental health conditions and symptoms before and after COVID-19 infection in individuals with long COVID. Understanding mental health-related conditions and symptoms prevalence prior to COVID-19 infection, in the period preceding long COVID diagnosis, and after the diagnosis can provide valuable insights into the association between mental health conditions and long COVID. As awareness of long COVID has increased, understanding the onset, progression, and underlying mechanisms has also become crucial for the development of treatment strategies and dealing with the consequences in those affected [25].

In this study, we aimed to investigate differences in the prevalence of depression, anxiety, fatigue, and headache a year prior to the COVID-19 pandemic (2019), 12 months prior to receiving a long COVID diagnosis, and 6 months after the diagnosis in individuals with and without a long COVID diagnosis.

## Methods

### Study design

A population-based case–control study design was used to examine the prevalence of mental health disorders and associated symptoms before and after the pandemic onset in individuals with and without long COVID in Region Stockholm, Sweden.

### Study context

The Swedish health system provides universal health coverage for all residents, and all necessary health care is publicly funded by regional- and municipal-level taxes and grants provided by the central government [26]. The regions determine co-payment rates and provider fees for all levels of care, including primary healthcare visits and hospitalizations. Services are provided by the region, either by regional-led service providers or by private providers with whom the region has a contractual agreement. Stockholm Region, which includes the city and surrounding suburban and rural areas, has approximately 2.5 million residents, making up approximately 25% of Sweden’s total population [27].

### The VAL database

Data was collected from the Stockholm Regional Health Care Data Warehouse (VAL databases), which is used for monitoring, quality assessment, planning, and remuneration of healthcare. The VAL databases contribute to national health registers and benchmarking and are utilized for research purposes. They contain detailed information on all registered diagnoses and healthcare visits within the region at primary, secondary, and tertiary care levels. The VAL databases have demonstrated their credibility through their contributions to national health registries and benchmarking reports [28].

### Study population

**Fig. 1:**
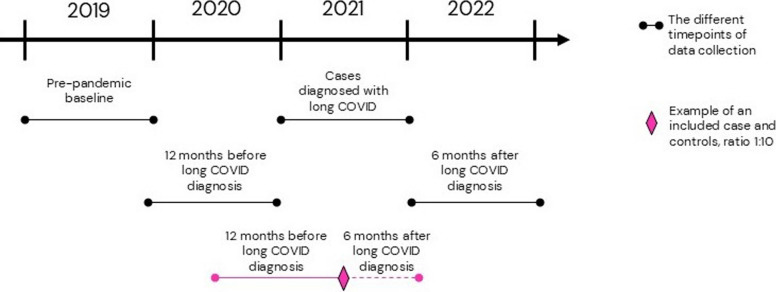
Outline of the data collection time points. Patients diagnosed with long COVID during 2021 (*n* = 5589) were matched 1:10 with non-long COVID controls (*n* = 47,561) based on age, sex, and socioeconomical neighborhood on the corresponding date. Data on depression, anxiety, fatigue syndrome after viral infection, and headache were retrieved for the timepoint of PASC diagnosis, a year prior to the COVID-19 pandemic (2019), 12 months prior to receiving long COVID diagnosis, and 6 months after the long COVID diagnosis

Cases comprised residents of Stockholm Region aged 18 and older, who were diagnosed with long COVID (ICD-10: U.09.9) between January 2021 and December 2021, but who were not hospitalized during the acute phase of COVID-19. The long COVID diagnosis was introduced in Sweden first on 16th of October 2020 [29], following the World Health Organization’s recommendations. The code was assigned by physicians in primary, secondary, and tertiary care settings, based on a history of confirmed or suspected COVID-19 infection and the presence of persistent symptoms lasting at least 12 weeks after initial infection, affecting one or more organs, and leading to functional impairment or reduced capacity in daily life, consistent with national guidelines at that time and later consensus on long COVID definition [30]. The cases were extracted for the period January 1–December 31, 2021, as illustrated in Fig. 1.

Each case was matched with 10 controls based on age, sex, and neighborhood socioeconomic status. Controls were assigned the study start date on the same day when respective cases obtained their long COVID diagnosis.

We collected the diagnoses registered during 2019 as well as diagnoses registered 12 months before and up to 6 months (a minimum of 40 days) after long COVID diagnosis for each case and the 10 matched controls without long COVID. The decision to collect diagnoses 6 months after long COVID diagnosis instead of 12 months was made due to our access to data from the VAL databases being limited to February 10, 2022.

### Collected data

The data collected included information about the individual, such as age, sex, ICD-10 diagnoses (depression F32, anxiety F41, and symptom-related diagnoses fatigue syndrome after viral infection G93.3, headache R51.9) [6, 31], and data related to neighborhood socioeconomic status. The Mosaic tool was utilized to categorize socioeconomic status into high, medium, and low [32, 33].

### Statistical analyses

The occurrence of specified diagnoses in cases versus controls was assessed using conditional logistic regression to compute odds ratios (OR) along with 99% confidence intervals (CI), to reduce the chance of spurious findings, accounting for multiple comparisons. Data processing and statistical analysis were conducted using SAS software, version 9.4 (SAS Institute Inc., Cary, NC, USA).

### Ethical approval

Ethical approval to use the register data without informed consent was granted by the Swedish Ethical Review Authority under act number 2021–01016 and later amendments 2021–05735-02.

## Results

### Study population characteristics

In total, 5589 patients with long COVID were included. Of those, 3862 were women (69%; mean age, 47 ± 12 years), and 1727 were men (31%; mean age, 47 ± 13 years). The cases were matched with 47,561 controls, where 32,151 were women (68%; mean age, 47 ± 12 years) and 15,410 were men (32%; mean age, 47 ± 13 years). The frequency of studied diagnoses registered during 2019, 12 months before, and 6 months after the long COVID diagnosis can be seen in Table 1 for women and in Table 2 for men.

**Table 1:** The frequency of all studied diagnoses in the year 2019, 12 months before, and 6 months after the long COVID diagnosis among women

Women	2019		12 months before the diagnosis		6 months after the diagnosis	
**Diagnoses ICD-10**	Frequency for cases, *n* = 3862 *N* (%)	Frequency for controls, *n* = 32,151 *N* (%)	Frequency for cases, *n* = 3862 *N* (%)	Frequency for controls, *n* = 32,151 *N* (%)	Frequency for cases, *n* = 3862 *N* (%)	Frequency for controls, *n* = 32,151 *N* (%)
Depression F32	162 (4.19%)	870 (2.71%)	140 (3.63%)	617 (1.92%)	87 (2.25%)	335 (1.04%)
Anxiety F41	357 (9.24%)	1873 (5.83%)	445 (11.52%)	1599 (4.97%)	287 (7.43%)	948 (2.95%)
Fatigue syndrome after viral infection G93.3	12 (0.31%)	51 (0.16%)	318 (8.23%)	76 (0.24%)	210 (5.44%)	59 (0.18%)
Headache R51.9	184 (4.76%)	645 (2.01%)	334 (8.65%)	641 (1.99%)	180 (4.66%)	295 (0.92%)
COVID-19, virus identified U07.1	X	X	900 (23.3%)	342 (1.06%)	139 (3.6%)	175 (0.54%)
COVID-19, virus non-identified U07.2	X	X	463 (11.99%)	297 (0.92%)	51 (1.32%)	34 (0.11%)
COVID-19 in own medical history, unspecified U08.9	X	X	312 (8.08%)	73 (0.23%)	279 (7.22%)	83 (0.26%)

**Table 2:** The frequency of all studied diagnoses in the year 2019, 12 months before, and 6 months after the long COVID diagnosis among men

Men	2019		12 months before diagnosis		6 months after diagnosis	
**Diagnoses ICD-10**	Frequency for cases, *n* = 1727 *N* (%)	Frequency for controls, *n* = 15,410 *N* (%)	Frequency for cases, *n* = 1727 *N* (%)	Frequency for controls, *n* = 15,410 *N* (%)	Frequency for cases, *n* = 1727 *N* (%)	Frequency for controls, *n* = 15,410 *N* (%)
Depression F32	36 (2.08%)	231 (1.50%)	47 (2.72%)	154 (1%)	40 (2.32%)	88 (0.57%)
Anxiety F41	97 (5.62%)	427 (2.77%)	136 (7.87%)	387 (2.51%)	89 (5.15%)	243 (1.58%)
Fatigue syndrome after viral infection G93.3	2 (0.12%)	8 (0.05%)	99 (5.73%)	22 (0.14%)	61 (3.53%)	7 (0.05%)
Headache R51.9	46 (2.66%)	144 (0.93%)	95 (5.50%)	140 (0.91%)	51 (2.95%)	94 (0.61%)
COVID-19, virus identified U07.1	X	X	336 (19.46%)	173 (1.12%)	50 (2.90%)	86 (0.56%)
COVID-19, virus non-identified U07.2	X	X	167 (9.67%)	100 (0.65%)	13 (0.75%)	18 (0.12%)
COVID-19 in own medical history, unspecified U08.9	X	X	144 (8.34%)	24 (0.16%)	108 (6.25%)	37 (0.24%)

### Occurrence and association of depression, anxiety, fatigue, and headache in individuals with long COVID

**Fig. 2:**
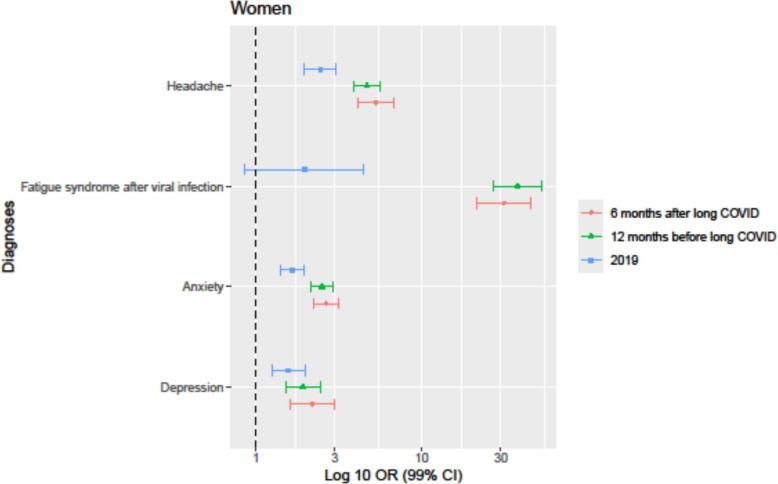
Odds ratios (log10 scale, 99% CI) for depression, anxiety, fatigue syndrome after viral infection, and headache among women with long COVID, a year prior to the COVID-19 pandemic (2019), 12 months prior to receiving long COVID diagnosis, and 6 months after the long COVID diagnosis

**Fig. 3:**
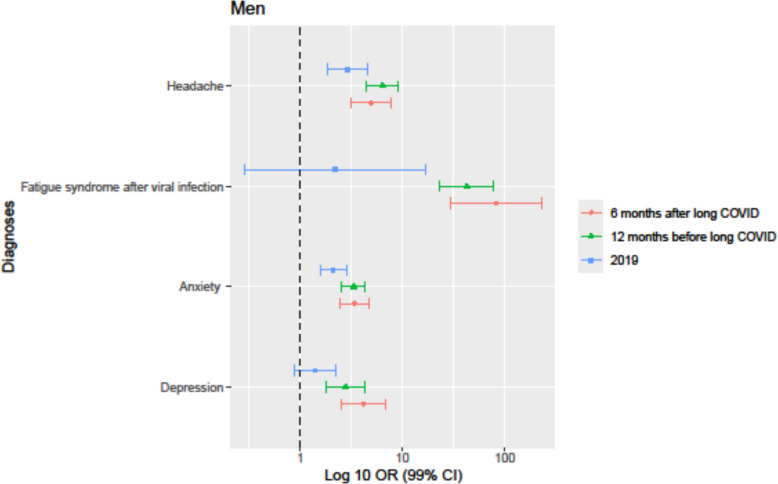
Odds ratios (log10 scale, 99% CI) for depression, anxiety, fatigue syndrome after viral infection, and headache among men with long COVID, a year prior to the COVID-19 pandemic (2019), 12 months prior to receiving long COVID diagnosis, and 6 months after the long COVID diagnosis

Long COVID diagnosis was significantly associated with a higher frequency of the studied diagnoses, *depression*, *anxiety*, and *headache* both before the start of the COVID-19 pandemic in 2019, 12 months before, and 6 months after long COVID diagnosis in comparison to matched controls, whereas *fatigue syndrome after viral infection* showed a significant association with long COVID in the 12 months before and 6 months after long COVID diagnosis only. The diagnoses *fatigue syndrome after viral infection* (except in 2019) and *headache* had the highest significant association with the incidence of long COVID across all time points and in both sexes, as shown in Tables 3 and 4 and illustrated in Figs. 2 and 3.

**Table 3:** The association between long COVID and relevant diagnoses in women shown as OR and 99% CI in the year 2019, 12 months before the long COVID diagnosis, and 6 months after the long COVID diagnosis

Women	2019	12 months before diagnosis	6 months after diagnosis
**Diagnoses ICD-10**	Odds ratios (99% CI)	Odds ratios (99% CI)	Odds ratios (99% CI)
Depression F32	1.57 (1.26–1.97)	1.92 (1.51–2.46)	2.19 (1.60–3.00)
Anxiety F41	1.65 (1.41–1.93)	2.50 (2.16–2.89)	2.65 (2.22–3.17)
Fatigue syndrome after viral infection G93.3	1.96 (0.86–4.48)	37.86 (27.16–52.77)	31.27 (21.34–45.82)
Headache R51.9	2.45 (1.96–3.05)	4.67 (3.90–5.59)	5.29 (4.13–6.78)

**Table 4:** The association between long COVID and relevant diagnoses in men shown as OR and 99% CI in the year 2019, 12 months before the long COVID diagnosis, and 6 months after the long COVID diagnosis

Men	2019	12 months before diagnosis	6 months after diagnosis
**Diagnoses ICD-10**	Odds ratios (99% *CI*)	Odds ratios (99% *CI*)	Odds ratios (99% *CI*)
Depression F32	1.40 (0.88–2.23)	2.79 (1.80–4.30)	4.15 (2.53–6.81)
Anxiety F41	2.10 (1.56–2.84)	3.33 (2.55–4.34)	3.41 (2.46–4.72)
Fatigue syndrome after viral infection G93.3	2.22 (0.29–17)	42.56 (23.11–78.40)	81.41 (29.06–228.10)
Headache R51.9	2.89 (1.86–4.50)	6.35 (4.48–9.00)	4.94 (3.14–7.77)

There was a higher frequency of all studied diagnoses 12 months before and 6 months after long COVID diagnosis compared with 2019. In particular, the odds ratio of *fatigue syndrome after viral infection* in patients with long COVID steeply increased from 1.96 (2019) to 37.86 (12 months before long COVID diagnosis) and 31.27 (6 months after long COVID diagnosis) for women, as shown in Table 3, and from 2.22 to 42.56 and 81.41, respectively, for men, as shown in Table 4.

At 6 months after long COVID diagnosis, the odds ratios for *fatigue syndrome after viral infection* in women and for *headache* in men were lower in comparison to 12 months before long COVID diagnosis; however, individuals with long COVID did still have significantly higher odds ratios in comparison to before the pandemic. Notably, for women, the odds ratio for *fatigue syndrome after viral infection* was lower at 6 months after long COVID diagnosis in comparison to 12 months before long COVID diagnosis, as shown in Table 3 and illustrated in Fig. 2. In contrast, for men, the odds ratio for *fatigue syndrome after viral infection* was higher at 6 months after long COVID diagnosis in comparison to 12 months before long COVID diagnosis, as shown in Table 4 and illustrated in Fig. 3.

## Discussion

In this study, we investigated whether individuals diagnosed with long COVID differed from matched controls in the occurrence of depression, anxiety, fatigue, and headache across three periods: 1 year prior to the COVID-19 pandemic (2019), 12 months prior to long COVID diagnosis, and 6 months after the diagnosis. Our findings clearly show that individuals with long COVID demonstrated a significantly higher frequency of depression and anxiety, as well as post-viral fatigue and headache, in comparison to matched controls before the COVID-19 pandemic in 2019, as well as 12 months before and 6 months after the long COVID diagnosis. All diagnoses were significantly associated with long COVID across all assessed periods, except for post-viral fatigue in 2019 and depression in men in 2019. Our findings indicate that the mental health burden in both sexes who later develop long COVID was already elevated before the pandemic in 2019, remained elevated 12 months prior to diagnosis, and continued 6 months after the long COVID diagnosis, compared with matched controls unaffected by long COVID controls.

About two-thirds of individuals diagnosed with long COVID in our study population were women. These findings are in line with existing literature on long COVID being more prevalent among women [14, 34] and the growing evidence that female sex is a risk factor for the development of long COVID [35]. Fluctuations in estrogen and progesterone may modulate immune and stress responses, potentially explaining increased vulnerability among women during certain life stages [36]. Sex differences in autonomic nervous system activity, including vagal tone and heart rate variability, may partly underlie observed disparities in symptom expression [37]. Recent evidence suggests that preexisting depression at the beginning of the pandemic may partly account for the higher prevalence of long COVID among women [38], complementing our findings of sex differences in mental health-related diagnoses. More broadly, sex differences in the prevalence and mechanisms of anxiety and depression [39] and in myalgic encephalomyelitis/chronic fatigue syndrome (ME/CFS) [40] may help explain our findings, underscoring the importance of systematically studying sex differences in related conditions [41].

Our findings, showing a higher prevalence of depression, anxiety, and post-viral fatigue and headaches in individuals diagnosed with long COVID compared to matched controls even prior to the COVID-19 pandemic, extend and refine knowledge from several recent studies. Using comprehensive healthcare register data allowed us to closely follow how the frequency of depression, anxiety, fatigue, and headache developed over time, both before and after the timepoint where long COVID diagnosis was established.

In particular, our results corroborate and extend the findings from a US cohort study by Wang et al. [42], which demonstrated that pre-infection psychological distress, including depression and anxiety, was associated with an elevated risk of long COVID. In contrast to the study by Wang et al., our study pertains to a general population and included more individuals. Importantly, as the study by Wang et al. relied on self-reported data collected through surveys, our findings strengthen the evidence by showing that the association between depression and anxiety was more prevalent in individuals with long COVID using detailed clinical diagnoses collected from healthcare register data, offering a complementary and more objective perspective [42]. Moreover, our use of comprehensive, population-based register data covering a general population and a larger sample size strengthens the external validity and generalizability of the observed associations.

Our findings also complement those of Lak et al. [43], who recently studied a large Swedish cohort and showed that symptoms such as depression, anxiety, and fatigue were more common pre-pandemically in individuals later diagnosed with long COVID. While their study broadly addressed symptom burden, our work specifically focused on clinically diagnosed depression, anxiety, fatigue syndrome, and headache and traced these trajectories before and after diagnosis. Together, these findings strengthen the evidence that individuals who develop long COVID may already differ from controls in terms of symptomatology and healthcare use, with our study adding a particular focus on mental health-related diagnoses.

Our findings are also in line with results from another recent population-based Swedish cohort study that used the same regional register data and found that mental health disorders and healthcare use before the pandemic were associated with long COVID among nonhospitalized individuals [44]. While that study primarily characterized factors associated with long COVID, our study extends this work by focusing specifically on mental health-related diagnoses, such as depression and anxiety, and examining their temporal patterns in relation to a long COVID diagnosis. Further, our study adds a novel dimension by quantifying the relative difference in diagnosis prevalence between cases and matched controls over time, offering deeper insight into the trajectory of mental health burden both before and after long COVID diagnosis.

Our findings also build on the results from a matched cohort study using English healthcare data, which showed that individuals with long COVID had a higher frequency of healthcare contacts in 2019 compared to matched controls without long COVID diagnosis, indicating a higher symptom burden already in the pre-pandemic period [45]. This study adds to previous findings by specifically addressing mental health-related diagnoses and by quantifying these associations with long COVID. Together, these findings strengthen the argument that individuals with long COVID may represent a distinct clinical subgroup already at risk prior to SARS-CoV-2 infection.

Our study showed that fatigue syndrome after viral infection and headache exhibited the strongest associations across all time points in both sexes. While the odds ratio for fatigue syndrome after viral infection declined 6-month post-diagnosis in women, and for headache in men, both conditions remained significantly elevated compared to pre-pandemic levels. This finding highlights the persistent burden of long COVID-related symptoms and aligns with previous research indicating that these are commonly reported long COVID-related diagnoses [2, 46].

Compared to pre-pandemic levels, we observed a significant increase in the occurrence of fatigue syndrome following viral infection during the 12 months before, i.e., after the presumed COVID-19 infection, and 6 months after a long COVID diagnosis. Interestingly, this trend differed by sex: in women, the odds ratios for fatigue syndrome after viral infection decreased 6-month post-long COVID diagnosis, whereas in men they increased.

Several potential explanations can be proposed. The association between pre-pandemic depression, anxiety, and fatigue with long COVID is likely multifactorial, involving both biological and psychosocial factors. Both depression and anxiety have been associated with chronic systemic inflammation, characterized by elevated levels of inflammatory markers [47–49]. This chronic inflammation might create a residual predisposition to acute and chronic inflammatory diseases, serving as a key common pathway underlying both pre-pandemic mental health symptoms and the subsequent development of long COVID.

Chronic low-grade inflammation has been implicated in the pathophysiology of mental health conditions such as depression and anxiety [47–49]. Similarly, persistent inflammation is considered a contributing factor to the prolonged symptoms observed in long COVID. Prolonged symptoms could also be explained by the relationship between immune responses, neuroinflammation, autonomic dysfunction, and psychosocial factors that may create a predisposition for prolonged symptoms following SARS-CoV-2 infection [11, 50]. These shared pathways suggest that preexisting mental health conditions may interact with other pathophysiological mechanisms to increase susceptibility to long COVID, rather than being the sole explanation for its symptoms. It is important to emphasize that our study is observational and cannot establish causality. The associations between preexisting mental health conditions and long COVID may also reflect a reverse causation, in which early or undiagnosed long COVID symptoms exacerbate mental health conditions, or confounding from factors such as chronic somatic illness or psychosocial stressors. These alternative explanations underscore the complexity of the observed associations and the need for cautious interpretation.

The similarities between long COVID, ME/CFS, and dysautonomia are worth mentioning [51]. Individuals with ME/CFS and dysautonomia are frequently assigned psychiatric diagnoses prior to receiving their current diagnosis, reflecting the diagnostic uncertainty and the complexity involved in distinguishing between these conditions in clinical practice [52].

A critical issue in the assessment of mental health diagnoses in long COVID lies in the tools used for evaluation. Many standard mental health screening instruments, such as anxiety and depression scales, incorporate physiological symptoms that overlap with autonomic dysfunction and post-viral fatigue. For instance, tachycardia, a common feature of dysautonomia, can contribute to inflated scores on anxiety scales, while profound fatigue, a hallmark of ME/CFS and long COVID, may be misinterpreted as a symptom of depression. The failure to account for these overlaps can lead to overestimations of mental health diagnoses in long COVID [53]. Further, it has also been debated whether there is a functional component in long COVID [20, 21], but our data can neither confirm nor refute this hypothesis. These multidirectional considerations emphasize the need for a nuanced approach in distinguishing between physiological and psychiatric components of long COVID, ensuring that patients receive appropriate and targeted care.

Our findings highlight the importance of continued research into the underlying mechanisms and pathophysiology of long COVID. While we observed a higher prevalence of depression, anxiety, fatigue, and headache in individuals with long COVID, both before and after diagnosis, it is crucial to recognize that these associations may be driven by complex interactions between biological, immunological, and psychosocial factors. For example, the sex-specific differences in the trajectory of symptoms such as post-viral fatigue and headache point to potential differences in immune or hormonal responses that warrant further investigation. Furthermore, we have shown in a parallel study on the same cohort that the post-COVID group had a significantly higher prevalence of respiratory diagnoses and symptoms, including asthma, prior to the pandemic (Lindberg P. et al., under review). Respiratory disease, as well as cardiac disease, may contribute to anxiety and depression and may thus act as a confounder in the assessment of mental health.

### Strengths and limitations

A major strength of the study was the use of the extensive population-based cohort and detailed healthcare data. Additionally, matching cases and controls by age, sex, and socioeconomic area reduces potential confounding bias. However, it is important to consider that some individuals with pre-existing mental health diagnoses are potentially more likely to seek medical care, increasing the likelihood of both receiving a long COVID diagnosis and having related symptoms documented. On the other hand, a history of psychiatric illness can bias clinicians toward attributing current symptoms to mental health issues, whereas in patients without such a history the opposite may occur. While we did not quantify this risk separately, the frequencies of depression, anxiety, fatigue, and headache provide an estimate of the baseline prevalence of these conditions prior to the pandemic. Distinguishing between pre-existing and de novo diagnoses is clinically important, as the emergence of new-onset conditions after COVID-19 infection may provide a stronger indication of long COVID-related pathology. In our study, we partly addressed this by presenting the frequency of depression, anxiety, fatigue, and headache in 2019 (pre-pandemic) as a baseline measure, which allows for comparison with the periods preceding and following a long COVID diagnosis. This approach provides an indirect estimate of potential new-onset cases. A large retrospective cohort study suggests increased psychiatric diagnoses following COVID-19 [54], but they often do not adjust for prior mental health status, potentially inflating estimates. Although quantifying this bias precisely is not possible with our data, another potential factor that could affect our results is the risk of underreporting of the long COVID diagnosis in the control group due to diagnostic challenges or limited access to primary health care. Another potential limitation of our study is related to the three time periods analyzed. While the 2019 period likely reflects a pre-COVID-19 and pre-long COVID baseline, the 12-month period prior to diagnosis may include individuals who were already infected with SARS-CoV-2 or experiencing early symptoms of long COVID.

While this study provides valuable insights into the relationship and temporal dynamics between long COVID and mental health diagnoses and symptom-related diagnoses, some limitations must be considered when interpreting the results. The retrospective design inevitably leads to the risks of misclassification bias and unmeasured confounders that might have influenced potential associations. However, the VAL databases offer data from prospectively registered electronic health data, which limits the information bias regarding the collected diagnoses. Consideration needs to be given to the ICD-10 code G93.3, which may encompass both post-viral fatigue syndrome and chronic fatigue syndrome (ME/CFS), potentially introducing diagnostic overlap. However, during the study period, most new G93.3 diagnoses in Sweden were issued in the context of post-viral conditions, including post-COVID-19, whereas established ME/CFS cases are relatively few in the population. Therefore, while some overlap cannot be excluded, it is unlikely to substantially affect the overall findings or conclusions of our study. Unfortunately, we were not able to perform a sensitivity analysis to explore this potential overlap due to data limitations. In this context, the elevated odds ratios for fatigue syndrome observed post-diagnosis may be interpreted in light of the fact that they could partly reflect low baseline prevalence in controls, diagnostic overlap with long COVID, surveillance bias, and shared pathophysiological pathways. Considerations need to be made regarding the unique circumstances surrounding the COVID-19 pandemic. The healthcare system underwent significant challenges and adaptations that might have affected clinical guidelines, procedures, and treatments. The healthcare-seeking behavior of the population, together with limited access to health care and fewer physical visits to primary care, needs to be considered when interpreting the results of our study. For example, there is a potential risk of including people with undiagnosed long COVID in the case–control group.

### Implications and future directions

Understanding the significance of preexisting health conditions is important in assessing the risk and prognosis of long COVID. Identifying individuals at higher risk of developing long COVID-related complications can inform early and targeted interventions and support strategies. However, our findings also emphasize the need for continued research into the biological and immunological mechanisms underlying long COVID. Likewise, psychological mechanisms may also play a role and should be considered in future research [55]. A deeper understanding of these mechanisms is essential for developing effective treatment strategies and providing appropriate care for those affected by long COVID. Importantly, our results support the hypothesis that individuals who develop long COVID may represent a distinct clinical phenotype characterized by an increased burden of mental health-related symptoms, both before and after infection with SARS-CoV-2.

The rise in odds ratios 12 months prior to long COVID diagnosis suggests that early recognition of symptom patterns could facilitate timely diagnosis and intervention. The high prevalence and strong association of depression, anxiety, fatigue syndrome after viral infection, and headache with long COVID highlight the need for multidisciplinary care approaches that address both physical and mental health. Practical strategies include routine screening for these symptoms in long COVID clinics; multidisciplinary case reviews involving primary care, neurology, rehabilitation, and mental health specialists; and timely referral to appropriate services. It is crucial that these approaches are informed by a thorough understanding of the pathophysiology of long COVID, rather than attributing symptoms solely to preexisting mental health conditions.

While our findings have important implications for clinical practice, they also raise several questions that warrant further investigation. For example, longitudinal studies with extended follow-up are needed to clarify how mental health trajectories evolve beyond 6 months and to determine the timing of symptom onset in relation to SARS-CoV-2 infection and long COVID diagnosis. Dose–response analyses could assess whether the severity or persistence of pre-pandemic depression and anxiety predicts long COVID risk. Mechanistic studies combining clinical data with biomarkers of inflammation, immune function, and autonomic regulation are also crucial for disentangling underlying pathways. Finally, larger sex-stratified analyses may help clarify the role of hormonal and immune factors in shaping differential outcomes between women and men.

## Conclusions

The present study shows that individuals who later developed long COVID were more likely than others to suffer from depression, anxiety, and headache before the COVID-19 pandemic. These findings underscore the importance of continued research into the underlying mechanisms and pathophysiology of long COVID, including the role of preexisting neuropsychiatric conditions and the relationship to other anxiety-generating conditions such as cardiac and respiratory disease. The higher prevalence of mental health-related diagnoses prior to long COVID may reflect preexisting conditions and differences in healthcare-seeking behavior rather than increased vulnerability alone. Understanding these mechanisms is essential for developing effective treatment strategies and providing appropriate care for those affected by long COVID. Future studies should aim to unravel the complex interplay between biological, immunological, and psychosocial factors to improve our understanding of long COVID and to guide evidence-based interventions.

## Data Availability

The data used in the present study are available for research purposes after ethical approval from Stockholm Region at halsodata. rst@regionstockholm.se.

## Abbreviations

COVID-19: Coronavirus disease 2019
SARS-CoV-2: Severe acute respiratory syndrome coronavirus 2
PASC: Post-acute sequelae of SARS-CoV-2 infection
Long COVID: Long-term effects of COVID-19
VAL: Stockholm Regional Health Care Data Warehouse
ICD-10: International Classification of Diseases, Tenth Revision
OR: Odds ratio
CI: Confidence intervals
ME/CFS: Myalgic encephalomyelitis/chronic fatigue syndrome
POTS: Postural orthostatic tachycardia syndrome
